# Retraction: Prevalence and public health significance of rabies virus in bats in the North Region of Cameroon

**DOI:** 10.1371/journal.pntd.0012171

**Published:** 2024-05-09

**Authors:** The PLOS Neglected Tropical Diseases Editors

Following publication of this article [1], a number of concerns were raised which question the scientific validity of the reported results. The concerns were discussed with two members of the *PLOS Neglected Tropical Diseases* Editorial Board who stated that:

- Existing literature [2, 3, 4, 5] indicates that detection of *Lyssavirus rabies* in bats in the West African nation of Cameroon is implausible.
- The direct immunofluorescence assay reported in Fig 2 alone is not sufficient to detect rabies virus as this assay cannot distinguish between different types of *Lyssavirus*.
- According to one member of the Editorial Board, the representative image provided for the positive detection of rabies ribo-nuclear protein in Fig 2B appears to contain microscopy artefacts and is therefore not sufficient as a positive control.
- The results of multiple studies [6, 7, 8, 9] referenced in this article are inaccurately reported, with estimates of prevalence reported as being derived from *Lyssavirus* antigen detection in bat brains as opposed to seroprevalence reporting of anti-lyssavirus antibodies in serum. Antigen detection and estimated prevalence in these studies was significantly lower than detection of neutralising antibodies.
- The risk assessment reported in the article does not meet required standards for prevention of human exposure to bat lyssavirus for which there is no rabies vaccine cross-protection.

The corresponding author responded to the above points, however the members of the *PLOS Neglected Tropical Diseases* Editorial Board concluded that these responses did not adequately address the concerns listed above.

In light of the unresolved concerns that question the reliability and validity of the reported results and conclusions, the *PLOS Neglected Tropical Diseases* Editors retract this article.

ID did not agree with the retraction. RSPN, MMMM, SDJ, RNGN, IC, LF, LGY, JMKF, AW, DM and JAN either did not respond directly or could not be reached.
